# YAP and TAZ Mediators at the Crossroad between Metabolic and Cellular Reprogramming

**DOI:** 10.3390/metabo11030154

**Published:** 2021-03-08

**Authors:** Giorgia Di Benedetto, Silvia Parisi, Tommaso Russo, Fabiana Passaro

**Affiliations:** Department of Molecular Medicine and Medical Biotechnology, University of Naples Federico II, 40, 80138 Napoli, Italy; giorgia.dibenedetto@unina.it (G.D.B.); silvia.parisi@unina.it (S.P.); tommaso.russo@unina.it (T.R.)

**Keywords:** YAP, TAZ, iPSC, cell reprogramming, metabolic reprogramming

## Abstract

Cell reprogramming can either refer to a direct conversion of a specialized cell into another or to a reversal of a somatic cell into an induced pluripotent stem cell (iPSC). It implies a peculiar modification of the epigenetic asset and gene regulatory networks needed for a new cell, to better fit the new phenotype of the incoming cell type. Cellular reprogramming also implies a metabolic rearrangement, similar to that observed upon tumorigenesis, with a transition from oxidative phosphorylation to aerobic glycolysis. The induction of a reprogramming process requires a nexus of signaling pathways, mixing a range of local and systemic information, and accumulating evidence points to the crucial role exerted by the Hippo pathway components Yes-Associated Protein (YAP) and Transcriptional Co-activator with PDZ-binding Motif (TAZ). In this review, we will first provide a synopsis of the Hippo pathway and its function during reprogramming and tissue regeneration, then we introduce the latest knowledge on the interplay between YAP/TAZ and metabolism and, finally, we discuss the possible role of YAP/TAZ in the orchestration of the metabolic switch upon cellular reprogramming.

## 1. Introduction

Cell reprogramming is the process of converting a somatic, specialized, cell into a different cell type. The term “reprogramming” can refer either to a direct conversion of a specialized cell into another or to a reversal of a somatic cell into an induced pluripotent stem cell (iPSCs) [1]. In any case, cell reprogramming implies a dramatic re-set of the whole cell, starting from the erasure and re-establishment of epigenetic marks acquired during development [2]. Reprogramming modifies the gene regulatory network of the resident cell type, to better fit the characteristics of the incoming target cell type, and this ultimately leads to changes in cell behavior, proliferation and metabolic rate, allowing the acquisition of a new phenotype [1]. The same changes in cell fate specifications can also be associated with some pathological cellular states. Indeed, tumorigenesis requires the reprogramming of a specific cell type into a new one with different characteristics in terms of gene expression, proliferation and metabolism and several types of tumors further dedifferentiate to acquire features different from those of the initial tumor mass [3,4]. As such, the somatic state is plastic, and can be modified by the right set of extracellular and intracellular signals.

Cellular reprogramming also implies an essential metabolic rearrangement, especially upon transition to induced pluripotency [5,6]. Somatic cells are often quiescent and their metabolism relies on oxidative phosphorylation (OXPHOS), whereas embryonic stem cells (ESCs) change their cell metabolism in diverse ways to meet the requirements for proliferation, gaining energy by increasing glucose conversion into lactate, even in oxygen-rich conditions [5,6]. This “aerobic glycolysis”, also named the Warburg effect from its discoverer Otto Warburg [7], depends on an increase in glucose uptake and highly activated glycolysis, and was originally employed to describe the metabolic switch occurring upon tumor progression. In the last decade, several common characteristics between cancer cell transformation and reprogramming have been established, with particular emphasis on the connections between Epithelial-to-Mesenchymal Transition (EMT) and its reversal Mesenchymal-to-Epithelial Transition (MET) [8], energy metabolism [9], DNA methylation and histone modification [10]. Very recently, a multi-layered analysis of mouse iPSC generation revealed stage-specific interdependences between EMT and metabolism, controlling an intermediary pluripotent state that can be converted to naïve and primed states [11].

Thus, the induction of a reprogramming process requires a nexus of signaling pathways, integrating a range of local and systemic information to control a series of cellular features, and accumulating evidence points to the crucial role exerted by the Hippo pathway components Yes-Associated Protein (YAP) and Transcriptional Co-activator with PDZ-binding Motif (TAZ). As downstream effectors of the Hippo signaling cascade [12], YAP and TAZ are crucial players of various cellular processes regulating organ growth, cellular plasticity, cell proliferation and stem cell biology [13,14,15]. Their activity is tightly regulated by a variety of upstream inputs, including the energy status of the cell, the nutrient availability, the composition of the tissue microenvironment and architecture, and extrinsic biochemical signals [16].

In this review, we will first provide a synopsis of the Hippo pathway and its function during reprogramming and tissue regeneration, then we will introduce the latest knowledge of YAP/TAZ regulation and function in metabolism and, finally, we will discuss the possible role of YAP/TAZ in the orchestration of a metabolic switch upon cellular reprogramming.

## 2. The Mammalian Hippo Pathway

The Hippo pathway is an evolutionary conserved signal transduction pathway, identified for the first time in Drosophila through genetic screening of tumor suppressors involved in tissue growth [17,18]. It plays a crucial role in organ development, tissue regeneration, stem cell maintenance, and tumorigenesis [12,19]. The pathway is mediated by a cascade of kinases, which ultimately phosphorylate the downstream effectors YAP and TAZ to modulate their subcellular localization.

The human YAP gene is mapped at chromosome 11q22 and encodes a proline-rich phosphoprotein capable of binding to the SH3 domain of the proto-oncogene tyrosine-protein kinase Yes [20]. It is expressed throughout the entire development process and ubiquitously in a wide range of tissues [21]. The human TAZ gene is mapped at chromosome 3q23-q24 [22]. TAZ and YAP share homology in protein sequence, as they both possess a WW domain for protein–protein interactions, a coiled coil domain, a transcriptional activation domain, and a C-terminal PDZ motif. Unlike TAZ, YAP also features an additional WW domain, a SH3-binding motif and a N-terminal proline-rich domain. Both YAP and TAZ lack the DNA binding domain, and for this reason, once translocated to the nucleus, they must interact with transcription factors in order to regulate target gene expression [16].

### 2.1. The Hippo Kinase Cascade

**Figure 1:**
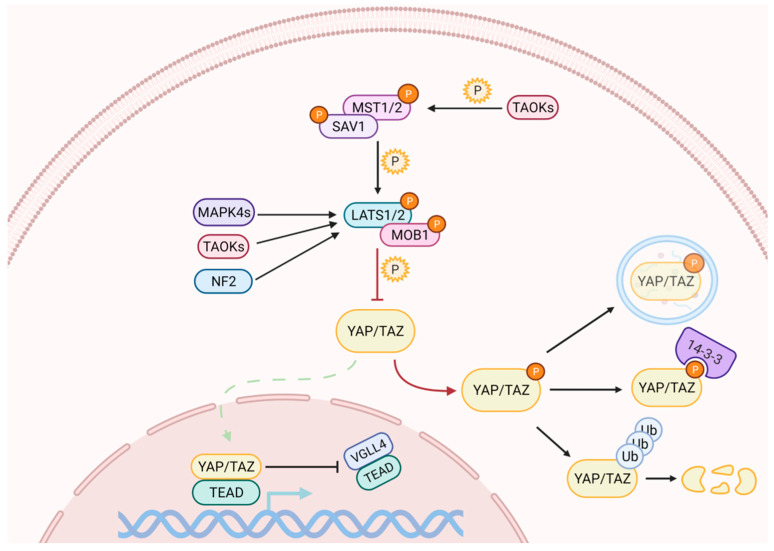
The Hippo signaling pathway comprises a cascade of phosphorylation and activation of kinases, which ultimately regulate YAP and TAZ subcellular localization. When the pathway is on, the activation of LATS1/2 triggers the inhibitory phosphorylation of YAP and TAZ. Phosphorylation on different residues may cause their cytoplasmic retention and/or their degradation through the ubiquitin-proteasome pathway. YAP can also be degraded by autophagy. When the pathway is off, YAP/TAZ dephosphorylation allows their nuclear import, where they compete with VGLL4 for binding to TEAD transcription factors to induce the expression of target genes.

Activation of the Hippo pathway prevents the nuclear translocation of YAP and TAZ (Figure 1). The kinase cascade begins with the dimerization of the Mammalian Sterile 20-like 1/2 (MST1/2, also named as STK4/3), which phosphorylates Salvador (SAV1) and MOB kinase activator 1A/B (MOB1A/B) to recruit Large Tumor Suppressor homolog 1/2 (LATS1/2). The subsequent phosphorylation of LATS1 on T1079 and LATS2 on T1041 is assisted by NF2, which interacts with LATS1/2 to bring it to the plasma membrane [23,24]. NF2, in its complexed form with KIBRA / WWC1 or Angiomotin (AMOT), can also induce LATS1/2 activation [25,26,27]. Subsequently, LATS1/2 undergoes autophosphorylation and activation [28]. In the absence of MST1/2, LATS1/2 can be activated through phosphorylation by proteins belonging to the MAPK4 family [29] and by proteins of the TAO kinase family [30]. The latter are also capable of stimulating indirect activation of LATS1/2, through the phosphorylation and activation of MST1/2 [31,32].

Active LATS1/2 can phosphorylate YAP and TAZ at multiple sites. The phosphorylation on YAP S127, and on TAZ S89, generates the binding sites for the protein 14-3-3, which holds them in the cytoplasm, preventing their nuclear translocation [33]. Further phosphorylation of YAP and TAZ on S381 and S311, respectively, generates the binding sites for the Casein1 kinase, which induces their degradation through the ubiquitin-proteasome pathway by recruiting SCF [34,35]. YAP can also be degraded by autophagy [36].

When the Hippo pathway is off, or upon occurrence, YAP/TAZ inhibitory phosphorylation can be rapidly reversed by phosphatases, resulting in their nuclear import [37,38], where they compete with Vestigial Like family member 4 (VGLL4) for binding to the transcriptional partner Transcriptional Enhancer factor Domain family members (TEAD) [39] to induce the expression of target genes. Although the TEAD family is among the main mediators [40], YAP and TAZ work synergistically with other transcription factors such as RUNX1/2 and SMAD family members [41,42].

### 2.2. Numerous Initiating Signals May Regulate YAP and TAZ Activity

A wide range of upstream inputs regulate the Hippo signaling, many of which relate to the cells’ local physical environment [12]. As such, YAP/TAZ regulation represents the final step of complex networks that integrate multiple factors, including biochemical and biomechanical cues [16]. Cell density, mechanical strength, cell adhesion, hypoxia, energy stress, endoplasmic reticulum (ER) stress, osmotic stress, and different growth factors are only a few examples of the possible modulators affecting YAP/TAZ activation [12]. Altered cell shape, ECM stiffness, cell stretching, cell density, and shear forces are all conditions associated with high levels of actomyosin contractility, which generally correlate with high YAP activity, whereas conditions associated with low actomyosin contractility are associated with low YAP activity [43,44].

Mounting evidence demonstrates that a number of other signaling pathways are able to converge on MST1/2 and/or LATS1/2 to regulate YAP/TAZ, and by now most major signaling pathways have been found to regulate or cross-talk with Hippo signaling. Protein kinases, including SRC kinase, protein kinase A (PKA), partition-defective or microtubule-affinity regulating (PAR-1/MARK) kinase, and TAO kinase (TAOK) are involved and participate in the initiation of the Hippo signaling [45,46]. Several growth factor receptors [47,48,49], the Wnt pathway [50,51] and many G Protein-Coupled Receptor (GPCR) signaling pathways [52] have been shown to interact with Hippo to modulate the activity of YAP/TAZ. Among these signals, many of them collaborate with each other, whereas others may be in conflict. How each cell selectively sorts out which signal(s) to respond to and/or converge all signals, or ignore and terminate them, remains a critical issue to resolve.

Finally, integration of growth and cell fate decisions also occur through cross-talk with metabolic pathways, and several connections between Hippo signaling and nutrient sensing and metabolic pathways have been identified and will be further discussed later in this review.

In summary, YAP/TAZ activation–inactivation is a dynamic process that integrates multiple cellular and extracellular inputs through different molecular mechanisms, which are all potential targets to modulate YAP/TAZ activity by pharmacological interventions.

## 3. YAP and TAZ in Tissue Regeneration and Cell Reprogramming

Adult human organs retain little or no potential to regenerate, thus tissue damage triggered by trauma, ageing or disease ultimately leads to organ disfunction [53].

In some tissues, the presence of resident, adult, stem cells (ASCs) ensures the turnover of old or damaged cells with newly differentiated ones [54]. In other tissues, like in the liver, regeneration is endowed by the ability of hepatocytes to reactivate cell proliferation [55].

Regenerative medicine aims at stimulating and enhancing such repair mechanisms directly in the site of tissue damage, in order to promote tissue repair, and the dissection of molecular effectors of tissue growth and regeneration will ultimately lead to novel potential therapeutic strategies.

The Hippo pathway plays a fundamental role in the regulation of stem cell biology, cell fate decisions, cell survival, differentiation, and de-differentiation and mounting evidence supports the requirement of YAP and TAZ for regeneration of different organs [13].

Nevertheless, YAP/TAZ seem to have pleiotropic and tissue-specific functions, promoting the growth of some organs, but supporting cell fate specification or stemness in others.

### 3.1. YAP and TAZ Promote Tissue Growth and Cell Specification during Development

During embryo development, the activity of the Hippo pathway components, in particular of the core kinases, is clearly required to restrict YAP/TAZ nuclear translocation, thus preventing aberrant tissue overgrowth. Indeed, loss of function of the core kinases or hyperactivation of YAP/TAZ in developing mice or of Yorkie in developing *D. melanogaster* causes overgrowth of multiple organs [13,14,15].

Nonetheless, YAP/TAZ functions during development extend beyond proliferation and tissue growth control [13], displaying crucial roles in the modulation of complex phenomenon such as cell fate specification and differentiation.

Homozygous YAP mutant mouse embryos die at embryonic day 8.5, showing defects in extraembryonic tissues, in body axis extension and neuroepithelium formation [21]. Homozygous TAZ mutant mice, instead, show no defects during embryogenesis, whereas adult animals develop pulmonary and kidney diseases [56,57]. Interestingly, the double knockout of YAP and TAZ dies before the morula stage, suggesting functional redundancy of these genes during early embryonic development [58].

To investigate YAP/TAZ functions at later stages of development and differentiation, conditional knockout mice have been generated. In developing heart, YAP deletion inhibits cardiomyocyte proliferation, causing cardiac hypoplasia [59,60].

Deletion of YAP in endothelial cells correlates with impaired angiogenesis and embryonic lethality [61]. During early development of coronary vasculature, YAP/TAZ inhibition causes the dysregulation of Tbx18 and Wt1 expression, impeding epicardial EMT, epicardial cell proliferation and differentiation into coronary endothelial cells [62,63].

YAP/TAZ play crucial roles at various stages of neurogenesis during the development of the mammalian central and peripheral nervous systems. Several studies demonstrated that YAP regulates brain development and homeostasis by balancing between neural stem/progenitor cell expansion and differentiation into post-mitotic neurons and glia. At early stages of embryonic development, the activation of the Hippo pathway controls the number of neural progenitors within the developing mouse brain by blocking YAP/TAZ-driven hypertranscription [64]. YAP inactivation also limits the size of neural progenitor cell pools within the developing neural tube [65], whereas YAP and TEAD activation facilitate cell cycle progression of the neural progenitor population through induction of cyclin D1, while inhibiting differentiation by NeuroM suppression [66]. At later stages, instead, a reduction in YAP/TAZ activity is required for promoting progenitor differentiation into mature functional neurons [67,68].

Nephrogenesis is also regulated by YAP/TAZ, as YAP activation through mechanical stress transduced via Rho GTPase Cdc42 promotes normal nephrogenesis [69]. The subsequent deactivation of YAP/TAZ through phosphorylation is required for inducing progenitor differentiation into mature functional nephrons. Indeed, conditional LATS1/LATS2 knockdown in nephron progenitors of mice leads to disruption of nephrogenesis due to YAP constitutive activation [70].

The spatiotemporal pattern of YAP expression during embryonic liver development [71] suggests that YAP may be required for the initial proliferation of hepatoblasts, while subsequent hepatoblast differentiation and maturation into hepatocytes entails YAP downregulation. Surprisingly, YAP/TAZ deletion during liver development did not affect adult liver size but caused defects in bile duct differentiation [72].

Similarly, YAP and TAZ deletion did not perturb the development and homeostasis of adult intestine, but the stem cell compartment located at the base of normal intestinal crypts displayed high levels of YAP [51].

In summary, the key role of YAP/TAZ during early development seems to be the governance of the delicate balance between proliferation and differentiation of precursor cells within various tissues. This role has also been conserved in adulthood, by regulating stem cell-dependent homeostasis and regeneration of adult organs.

### 3.2. YAP and TAZ Promote Tissue Regeneration by Regulating Stemness, Dedifferentiation and Differentiation

The expression of YAP and TAZ is enriched in diverse stem cell populations in vivo and in vitro. In human embryos, for instance, YAP is localized to the nucleus of inner cell mass (ICM) cells of the developing blastocysts [73], which contain the ESCs. Conversely, in mouse embryos, YAP localized to the nucleus of trophoblast cells, which are stem cells that give rise to extra-embryonic tissues, and not to the ICM [58]. Both human and mouse ESCs display high levels of YAP when cultured in condition preserving pluripotency, although the possible role of YAP/TAZ in governing pluripotency is still controversial. Preliminary studies on in vitro mouse ESCs reported that YAP silencing induced a loss of pluripotency, while its ectopic expression hampered ESC differentiation [74]. Furthermore, YAP seemed to be a direct regulator of Octamer-binding transcription factor ¾ (OCT3/4) gene expression by interacting with TEAD2 [75]. Later, other studies pointed out that YAP and TAZ might be dispensable for mouse ESC pluripotency, but required to sustain the proper differentiation [50,76].

More recently, a new role of YAP has emerged as a regulator of the switch between pluripotency and differentiation of mouse ESCs. In support of this notion, there is evidence indicating that blocking YAP from contributing to the TEAD/β-catenin-TCF3 complex in mouse ESCs by the tumor suppressor RASSF1A is crucial for the onset of differentiation [77]. We have contributed to the field by demonstrating that YAP silencing induces a robust deregulation of de novo methylation events that usually guide the exit of ESCs from pluripotency upon differentiation [78].

Since self-renewal and pluripotency maintenance involves different mechanisms in human compared to mouse ESCs [79], findings with mouse ESCs may not necessarily be applicable to human ESCs. In fact, YAP/TAZ play a key role in maintenance of stem cell phenotype in human ESCs [80,81,82,83].

Besides ESCs, YAP/TAZ are also involved in the expansion, self-renewal and maintenance of stem cell phenotype of ASCs, including neural stem cells [84,85], muscle satellite cells [86], and intestinal stem cells [87], whose activation and mobilization is required to sustain normal tissue homeostasis and to support the regeneration process following tissue disease or injury.

YAP/TAZ are required to maintain proliferation of Transit-Amplifying cells and to inhibit their differentiation by a mechanism comprising the FAK-YAP-mTOR signaling axis [88]. In addition, self-renewal and differentiation of bone-marrow derived mesenchymal stem cells (MSCs) seem to be dependent on the interaction between YAP/TAZ and the zinc-finger transcription factors Snail/Slug [89]. Mechanosensing of substrate stiffness via YAP/TAZ regulates differentiation of MSCs into either the adipogenic or osteogenic lineage. Increases in cytoskeletal tension via Rho GTPase, focal adhesion kinase (FAK) and MAPK signaling pathways ultimately increases YAP/TAZ nuclear translocation, promoting osteogenesis [90], while adipogenic differentiation is promoted by decreased YAP/TAZ activity triggered by low cytoskeletal tension [91].

In agreement with embryonic neural development, enhanced differentiation of human neural stem cells has been reported upon YAP cytoskeletal sequestration [83,92].

YAP plays a crucial role in the cell fate determination of muscle satellite cells, which are stimulated to proliferate and differentiate into myoblasts upon muscle injury. Indeed, increased nuclear YAP translocation induces satellite cell activation into highly proliferative myoblasts [86]. However, further maturation into terminally differentiated myotubes require subsequent deactivation of YAP through phosphorylation [93].

Epidermal healing and regeneration are mediated primarily by a resident pool of adult stem cells located in the basal layer of the epidermis. YAP protein regulates the balance between epidermal stem cell proliferation and differentiation [94,95], thus representing a master player of skin regeneration. Mechano-activation of YAP/TAZ promotes epidermal stemness via inhibition of Notch signaling, whereas YAP/TAZ inhibition by weak mechanical forces induces Notch signaling and differentiation [96].

Proliferation and differentiation of intestinal stem cells, required for homeostasis and regeneration of the intestinal epithelium, also involves regulation of YAP/TAZ activity, with high levels of YAP/TAZ activation promoting proliferation and inhibiting differentiation, and TEADs and Kruppel-like factor 4 (KLF4) identified as YAP/TAZ transcriptional partners [87,97].

These results indicate that YAP/TAZ may serve as a switch between quiescence, proliferation and differentiation of ASCs. This specifically highlights the relevant contribution of YAP/TAZ to ASC-dependent tissue homeostasis and regeneration.

Nevertheless, there are tissues, like the liver and the heart, in which YAP/TAZ are largely dispensable for tissue homeostasis, but their activity is essential for regeneration upon injury. Several studies have reported increased YAP/TAZ activation in ischemic heart disease and dilated cardiomyopathy, suggesting a role in heart repair and regeneration [98], possibly by stimulation of cardiomyocyte proliferation. Indeed, heterozygous deletion of YAP significantly decreases cardiomyocyte proliferation and exacerbates injury in response to chronic myocardial infarction [99]. In the adult mouse liver, the expression of YAP is low or absent in hepatocytes, but upon liver injury, or after partial hepatectomy, YAP expression rapidly increases in regenerating hepatocytes. Moreover, YAP/TAZ activation is required for the mobilization and proliferation of hepatic stellate cells, the endogenous resident adult stem cell pool within the liver that contributes to regeneration following disease or injury [100].

Altogether, these results highlight the fundamental contribution of YAP/TAZ to tissue regeneration, although specific functions may differ in different organs. As such, YAP/TAZ are emerging as possible targets to guide tissue regeneration processes for future progress in tissue engineering and regenerative medicine applications.

### 3.3. YAP and TAZ Manipulation for Cellular Reprogramming

Studies on reprogramming of adult somatic cells into iPSCs suggested that the process may take advantage of YAP activation, although some conflicting results exist between humans and mice. YAP ectopic expression in human amniotic epithelial cells allowed the reprogramming to iPSCs by only two reprogramming factors—OCT4 and SRY (sex determining region Y)-box 2 (SOX2)—instead of the four OCT4, SOX2, MYC proto-oncogene (c-MYC) and KLF4 (OSMK) usually required [101]. Reprogramming of human somatic cells into iPSCs may also benefit from LATS1/2 knockdown and the consequent YAP nuclear translocation [102]. Moreover, sustained overexpression of YAP in human ESCs and iPSCs seems to promote the generation of naive pluripotent stem cells [73]. More recently, in contrast with previous results obtained in human somatic cells, a non-cell-autonomous function of YAP has been proposed to promote mouse pluripotency induction. Hartman et al. reported that co-expression of YAP together with OSMK potently inhibits the reprogramming process in a cell-autonomous manner, whereas co-culture of mouse embryonic fibroblasts (MEFs) overexpressing YAP with MEFs overexpressing OSKM greatly promotes the emergence of pluripotency in the latter in a paracrine fashion, by increasing the expression of secreted matricellular proteins, such as cysteine rich angiogenic inducer 61 (CYR61) [103]. The precise mechanism remains to be elucidated, but CYR61 modulates inflammation and senescence [104], both of which have been implicated in reprogramming induction via non-cell-autonomous mechanisms [105].

YAP/TAZ activation has also been associated with mouse somatic cell reprogramming into tissue-specific stem/progenitor cells. Transient expression of exogenous YAP or TAZ efficiently reprograms various lineages of primary differentiated mouse cells, such as mammary gland, neuronal, and pancreatic exocrine cells, to proliferative cells with progenitor-like properties [106].

Interestingly, YAP/TAZ also have a role in reprogramming of the intestinal epithelium into a primitive state during the in vivo regeneration process. During this process the suppression of marker genes of differentiated cells and the parallel de novo expression of fetal markers can be observed, and an in vitro experiment with a collagen 3D matrix supplemented with Wnt ligands to sustain endogenous YAP/TAZ activation recapitulate the reprogramming of cell fate [107].

Finally, YAP/TAZ hyperactivation could induce the transdifferentiation of mature hepatocytes into ductal cells bearing characteristics of hepatic progenitors [108].

Of note, not all reprogramming processes necessitate the activation of YAP/TAZ. Direct cardiac reprogramming generates cardiomyocytes from fibroblasts without passing through a stem cell state [109]. This transdifferentiation requires a massive epigenetic remodeling to allow the silencing of fibroblast gene programs and, in parallel, the upregulation of the transcriptional rate of genes in cardiogenic loci [110]. Very recently, it has been demonstrated that the use of soft matrices resembling the native myocardium improved cardiac reprogramming by suppressing YAP/TAZ signaling [111]. This is in line with other evidence suggesting that YAP/TAZ deactivation is usually associated with the restriction of heart growth at birth [60], that YAP deactivation is associated with a switch to fatty acid metabolism that in turn inhibits proliferation and promotes cardiomyocyte maturation in a cardiac organoid model [112], and that YAP deactivation leads to downregulation of polo-like kinase 2, which in turn enables cardiac progenitors to switch from the proliferative to the terminal differentiation phase [113].

In summary, this evidence suggests that the manipulation of YAP/TAZ activity might be a powerful tool for the induction of tissue repair and regeneration in different tissues. Nevertheless, the hyperactivation of YAP/TAZ may also give rise to safety concerns, as it can result in aberrant cell proliferation, fibrosis and tumorigenesis. Thus, whether and how YAP/TAZ can be activated safely and effectively in human patients still needs to be investigated.

## 4. YAP and TAZ Are Modulators of Metabolic Reprogramming

**Figure 2:**
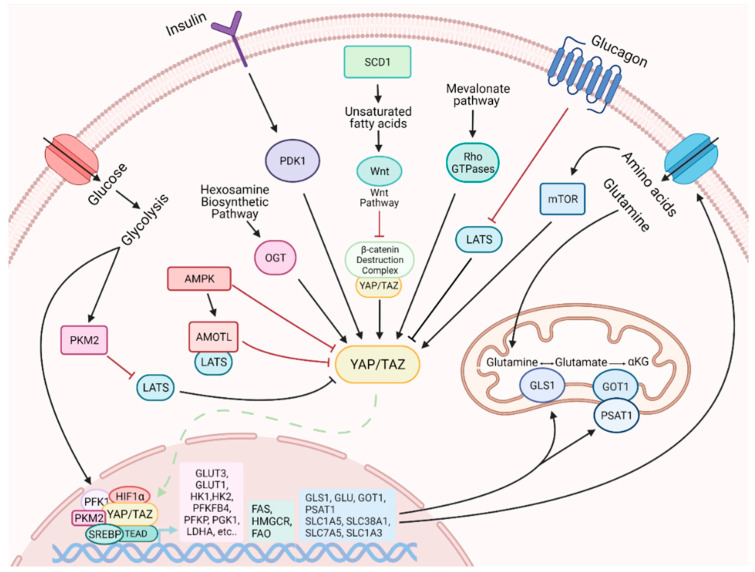
The activity of YAP/TAZ is influenced by various metabolic cues. Some of them can activate YAP and TAZ, such as high levels of glucose, the monosaturated fatty acid biosynthetic pathway and the mevalonate pathway, IIGF signaling and mTOR. Some others, like low glucose condition and glucagon stimulation, act by inactivating YAP/TAZ. YAP and TAZ, in turn, influence cell metabolism in various ways (see the text for more details).

Cellular reprogramming implies an essential metabolic rearrangement, necessary to face the changes in bioenergetic and biosynthetic demands [114]. Among the thousands of genes whose expression is modified upon reprogramming, many are directly or indirectly related to metabolism [5,6,115]. Our understanding of the role exerted by YAP/TAZ in regulating metabolism is rapidly expanding [116]. It is worth noting that not only may their activity be influenced by the cellular metabolic status, but they are also able to promote a metabolic remodeling process, which may accompany cellular adaptation to changes in the environmental conditions (Figure 2).

### 4.1. Metabolic Status Influences YAP and TAZ Activity

The activity of YAP/TAZ is influenced by various metabolic cues, such as glucose, lipids, amino acids and other metabolites with signaling activity. Multiple studies revealed that cellular glucose levels affect YAP/TAZ phosphorylation and inactivation [117,118,119], which likely plays a role in coordinating glucose/energy status with cell growth. A crucial role is exerted by the glycolytic enzyme phosphofructokinase (PFK1), which promotes YAP-mediated gene transcription by binding to TEADs [117]. When glucose levels are reduced and glycolysis rate decreased, PFK1 levels also decrease and YAP-mediated transcription is downregulated. Another crucial factor in the regulation of YAP activity is the AMP-activated protein kinase (AMPK) metabolic sensor [118,119,120]. A high AMP/ATP ratio activates AMPK, which in turn directly phosphorylates and inhibits YAP [117,118]. Furthermore, AMPK phosphorylates and stabilizes Angiomotin Like 1 (AMOTL1), and this promotes LATS activation and further YAP phosphorylation and inhibition [118].

The nutrient-sensitive hexosamine biosynthetic pathway, and in particular the key enzyme O-GlcNAc transferase (OGT), has also been shown to regulate YAP [121,122]. In elevated glucose level conditions, OGT catalyzes the O-GlcNAcylation on YAP S109, which promotes YAP expression, improves its stability, prevents its phosphorylation by LATS and activates its transcriptional activity [121,122]. Moreover, the O-GlcNAcylation on YAP T214 not only prevents the βTrCP-YAP interaction, which usually triggers YAP protein degradation, but also antagonizes LATS1 phosphorylation on YAP S127, promoting its transcriptional activity [119,120,121,122].

Lipid availability is a critical factor for efficient cell proliferation since lipids serve as precursors of cell membrane components. Highly proliferative cells exhibit a strong demand for lipids and cholesterol, which can be achieved by increasing either uptake of exogenous lipids or de novo synthesis [123]. The activity of YAP/TAZ is regulated by key enzymes involved in lipid metabolism. Stearoyl-CoA-desaturase 1 (SCD1), the enzyme that synthesizes monounsaturated fatty acids, promotes the activity of YAP and TAZ, in a mechanism partially dependent on the Wnt/β-catenin pathway [124]. Wnt ligands stimulate the synthesis of unsaturated fatty acids by activating SCD1, which mediates the release of β-catenin and YAP/TAZ from the β-catenin destruction complex [50]. Once released from the complex, β-catenin, YAP and TAZ translocate to the nucleus to perform their function. The mevalonate pathway is a lipid metabolic pathway that ensures the synthesis of sterols and other nonsteroidal lipids from acetyl-CoA. This pathway promotes YAP/TAZ activity through Rho GTPases, which require prenylation for their membrane localization and activity [125].

Finally, both insulin and glucagon pathways regulate YAP/TAZ activity. YAP protein is activated by insulin–IGF signaling (IIS), and YAP in turn is necessary for insulin-induced growth [49]. This occurs through PDK1, with additional contributions from AKT. Glucagon, instead, negatively affects YAP/TAZ activity by activation of LATS1/2 [52].

### 4.2. YAP and TAZ Influences Cell Metabolism

YAP and TAZ are now recognized to reprogram cellular metabolic pathways, allowing cells to adapt to mutable environmental conditions [116,126]. They integrate physiological and environmental signals by modulating transcriptional programs to regulate the expression of metabolic enzymes and nutrient transporters. They also take part in the regulation of biogenesis and the functioning of some intracellular organelles, in particular mitochondria [127]. However, most of the information on YAP/TAZ regulation of cellular metabolism derived from studies on tumor cells, in which they usually work as oncogenes and are involved in the promotion of the switch to aerobic glycolysis.

YAP/TAZ are involved in regulation of glucose metabolism [126]. In tumor cells, depletion of YAP and TAZ induced by silencing leads to a reduction in aerobic glycolysis-dependent growth, an increase in mitochondrial respiration and accumulation of reactive oxygen species (ROS), whereas the upregulation of glucose transporters and glycolytic key enzymes is one of the effects of YAP and TAZ activation [127]. In partnership with the hypoxia-inducible factor 1α (HIF1α), YAP regulates the expression of a number of aerobic glycolytic genes [128,129] to increase glucose absorption and the glycolysis rate. YAP promotes GLUT3 transcription via the TEAD responsive element conserved in the GLUT3 promoter [119]. In addition, GLUT3 expression can be stimulated by the interaction of YAP with the pyruvate kinase M2 (PKM2) enzyme [130]. YAP stimulates the expression of hexokinase 2 (HK2) at both the mRNA and protein levels [131]. In this action, FOXC2 seems to play a central role: in the nucleus, it interacts with YAP and TEAD for the formation of the FOXC2-YAP-TEAD complex to promote HK2 transcription and consequently glycolysis [131]. This mechanism is still controversial, unlike the YAP/TEAD/p65 complex that has been shown to bind to the HK2 promoter region and to promote glycolysis in breast cancer cells [132].

Interestingly, other studies in breast cancer revealed that YAP also regulates the glycolytic rate indirectly, by promoting the transcription of long non-coding Breast Cancer RNA Resistance to Antiestrogens 4 (BCAR4), which subsequently activates the Hedgehog effector GLI family zinc finger 2 (GLI2), to form a BCAR4/GLI2/p300 complex, which, through the acetylation of histones H3K27ac, induces HK2 and 6-phosphofrutto-2-kinase/fructose-2, 6-bisphosphatase 3 (PFKFB3) gene transcription [133].

Despite YAP/TAZ-mediated control of glucose metabolism being cell-type dependent and possibly requiring changes of multiple glycolysis enzymes rather than a single component, all these studies support a notion that high YAP activity alters cellular metabolic programs to enhance glucose uptake and utilization.

Regarding the active role of YAP and TAZ in lipid metabolism, it seems that their activity is tissue specific: they stimulate lipid accumulation, as in the liver [134], but they decrease lipid deposition, as in adipocytes of the brown adipose tissue [135]. It has been demonstrated that YAP interacts in the nucleus with the Sterol Rgulatory Element-Binding Proteins 1 and 2 (SREBP1 and SREBP2) and enhances their transcriptional activities for fatty acid synthase (FAS) and 30-hydroxylmethyl glutaryl coenzyme A reductase (HMGCR) [136]. Moreover, the activation of LATS1 or inhibition of YAP reduces hepatic steatosis and hyperlipidemia in diet-induced diabetic mice [136]. Meanwhile, in the process of metastasis of the lymph nodes, YAP induces a metabolic reprogramming by switching from glycolysis to fatty acid oxidation (FAO) for energy production [137].

Glutamine is a key amino acid involved in energy generation, biosynthesis of amino acids, nucleotides, and fatty acids, and control of redox state, and YAP/TAZ regulate glutamine metabolism in diverse way [138,139]. In the liver, YAP and TAZ stimulate glutamine synthetase to produce glutamine in order to favor the de novo nucleotide synthesis pathway [138], whereas in pulmonary arterial hypertension, vascular stiffness activates YAP/TAZ, which in turn promote not only glycolysis, but also glutaminolysis in pulmonary vascular endothelial cells, through the modulation of the glutaminase enzyme [140]. In breast cancer, YAP/TAZ guide a metabolic reprogramming to promote a close dependence of tumor cells on exogenous glutamine by inducing the expression of glutamic-oxaloacetic transaminase (GOT1) and phosphoserine aminotransferase (PSAT1) [141]. YAP and TAZ also upregulate different genes encoding high affinity transporters involved in the uptake of amino acids to increase their availability, such as Solute Carrier family members SLC1A5, SLC7A5, SLC38A1 and SLC1A3 [139,142].

The activation of mTOR is highly dependent on amino acids and can be regulated by YAP/TAZ. Several studies demonstrate a bidirectional regulation: on the one hand, YAP increases the activity of mTOR through the suppression of phosphatase and tensin homolog (PTEN) via microRNA [143], and on the other both mTOR Complexes mTORC1 and mTORC2 positively regulate YAP in perivascular epithelioid cell tumors and glioblastomas [36,144,145]. Finally, YAP/TAZ also has a great impact on mitochondrial homeostasis [146,147].

All these data not only confirm the essential role played by YAP/TAZ in the regulation of cellular metabolism, but demonstrate that metabolic changes in turn modulate YAP and TAZ activation, making these proteins essential players at the crossroad between metabolism and regulation of cell identity [116].

## 5. Potential Role of YAP and TAZ in the Orchestration of a Metabolic Switch upon Cell Reprogramming

Over the last decade, many studies have highlighted the importance of metabolic remodeling during reprogramming [5,6,114].

It is well known that metabolism has an active role in the regulation of cell identity and behavior during embryonic development, and recent studies demonstrate that metabolic remodeling is not only associated with reprogramming, but also appears to mediate the process [5,6,114].

**Figure 3:**
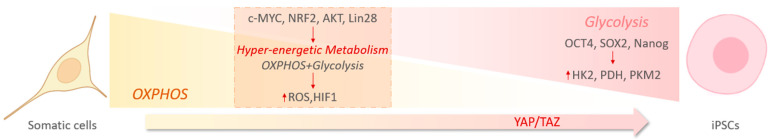
Cell reprogramming is accompanied by a decrease in OXPHOS and an increase in glycolysis. Shortly after reprogramming begins, cells undergo a transient hyperenergetic metabolism, which generates ROS, leading to the activation of HIF1, which further stimulates metabolic reprogramming together with c-MYC, NRF2, AKT and Lin28. A direct transcriptional regulation of glycolytic genes depends also on pluripotency factors OCT4, SOX2 and Nanog. YAP/TAZ activation may regulate the switch from OXPHOS to glycolysis in cell reprogramming, promoting glucose uptake and utilization, enhancing glutaminolysis, regulating the expression of metabolic genes, affecting epigenetic modifications to chromatin structure and post-transcriptional miRNA processing. Red arrows before ROS and HK2 mean “increased expression”.

All evidence supporting the involvement of YAP/TAZ in the metabolic remodeling of cancer cells [116] also suggest a possible role for these proteins in metabolic changes observed upon cellular reprogramming (Figure 3).

### 5.1. Mechanisms of the Metabolic Switch during Cell Reprogramming

The overall trend of the metabolic changes during reprogramming consists of decreasing OXPHOS and increasing glycolysis, as a main source of ATP production [5]. The switch is accompanied by alterations in the amounts of corresponding metabolites [6], which are shunted to various anabolic pathways. To maintain a high flux of metabolites in the glycolytic pathway, the key enzymes in this process including HK2, pyruvate dehydrogenase (PDH), and PKM2 are expressed at higher levels in iPSCs than in somatic cells. A direct transcriptional regulation of such genes depends on the core pluripotency factors, OCT4, SOX2, and Nanog, which occupy many regions of glycolytic enzyme genes [148].

Many studies have shed light on the molecular mechanisms that regulate the acquisition of this unique metabolic state during reprogramming. Genome wide analysis of gene and protein expression, as well as metabolomic profiling, confirmed that somatic cell reprogramming to iPSCs is initially accompanied by a massive activation of a pattern of genes necessary to increase the proliferation rate and to trigger those metabolic changes necessary to prepare the cell for new energy and metabolite demand arising during the subsequent steps of reprogramming [5,114]. The effect of the increasing expression of metabolism-related genes shortly after the start of reprogramming is that cells undergo a transient hyper-energetic metabolism, which is somewhat reminiscent of the metabolic state typical of naïve ESCs, in which high OXPHOS and high glycolysis co-exist [149,150] for a short period of time. This hyper-energetic state generates ROS, leading to activation of HIF1, which initiates a cascade of transcription factor induction that elicits the subsequent metabolic shift to a more glycolytic phenotype [150]. In addition to its well-known role in the maintenance and acquisition of stem cell properties and its relevance in metabolism of primed stem cell state [150,151,152,153], the crucial role of HIFs in the switch from oxidative to glycolytic metabolism during reprogramming is emphasized by the fact that its knockdown in human fibroblasts prevents reprogramming [151,152], and that reprogramming efficiency is enhanced in 5% oxygen compared with the standard 20% [153].

Other important factors playing a pivotal role in the acquisition of the transient hyper-energetic state are c-MYC [154], the nuclear factor, erythroid 2 like 2 (NRF2) [150], AKT [155] and OCT4 [148], which stimulate the metabolic flow of glycolysis by acting at a transcriptional and post-transcriptional level on the regulation of the key regulatory enzymes of the glycolytic pathway. In addition, the reprogramming factor Lin28, an evolutionary conserved RNA-binding protein that plays important roles during embryonic development and tumorigenesis [156,157,158] is involved in the repression of OXPHOS genes by binding to their mRNA [159].

Another distinct feature of the metabolic remodeling occurring in somatic cells upon reprogramming to iPSCs involves mitochondria that undergo a significant remodeling encompassing morphological and functional changes to adopt a “rejuvenated” state [5,6,114,160,161]. iPSCs appear to consistently have a low mitochondrial mass. Accordingly, the amount of mitochondrial DNA gradually decreases during reprogramming [162]. Mitochondria also revert to a more immature phenotype resembling the ESC-like state in terms of morphology, cellular distribution and efficiency of oxidative phosphorylation [163], in accord with the overall metabolic switch from mitochondrial oxidation to glycolysis. This reorganization entails the removal of mature mitochondria by mitophagy, an Atg5-independent selective autophagy, and the generation of new immature mitochondria [162]. The AMPK-mTOR signaling axis seems to play an essential role in this mitochondria clearance [162].

Reprogramming somatic cells to a pluripotent state requires the removal of somatic marks from the genome and the establishment of a pluripotent state and this occurs by modification of the epigenetic landscape and chromatin conformation, which in turn affects gene expression [164,165]. Metabolites directly prompt cell reprogramming by influencing the modification of epigenetic marks [166]. Chromatin modifications such as H3K9me3, H3K27me3 and DNA methylation function to maintain a somatic chromatin state that must be removed during somatic reprogramming, whereas other histone methylation marks, such as H3K4me3 or H3K4me1, are important to allow the proper transcription of pluripotency genes. In both ESCs and iPSCs, the histone methylation mark depends on threonine and S-adenosyl methionine (SAM) metabolism, where SAM is a substrate for methyl transferases [167]. SAM is present at high levels in iPSCs [168] and its depletion leads to reduced H3K4me3 marks and defects in the maintenance of the ESC state, as well as in the key signaling pathways important for the metabolic changes [169]. The histone acetylation mark functions to keep the chromatin relaxed in order to be transcribed, and this mechanism depends on the amount of acetyl-coenzyme A (acetyl-CoA) produced by the cell [167,170]. During reprogramming, not all the glucose is converted to lactate, because it is also used to produce acetyl-CoA by the PDH enzyme [170]. The acetyl-CoA is converted to citrate by citrate synthase in the mitochondria. Then, citrate is exported to the cytoplasm where it is again converted to cytosolic acetyl-CoA by ATP-citrate lyase. Cytosolic acetyl-CoA is required for histone acetylation to maintain the open state of the chromatin structure [170,171]. In contrast, the loss of acetyl-CoA results in histone deacetylation and loss of pluripotency in iPSCs [170].

In summary, the efficiency of reprogramming depends on the modulation of metabolism, and conversely, when the reprogramming efficiency is altered, it is usually accompanied by an altered metabolism. This suggests that this metabolic shift and all the molecules involved in its regulation might play an active role in this process.

### 5.2. YAP and TAZ in the Metabolic Remodeling of Cell Reprogramming

YAP/TAZ are now recognized to reprogram cellular metabolic pathways allowing cells to adapt to mutable environmental conditions [126]. They integrate physiological and environmental signals by modulating transcriptional programs to regulate the expression of metabolic enzymes and nutrient transporters. They also take part in the regulation of biogenesis and the functioning of some intracellular organelles, in particular mitochondria [126,146,147]. However, most of the information on YAP/TAZ regulation of cellular metabolism is derived from studies on tumor cells [116], in which they usually work as oncogenes, while only a few studies have explored the possible role exerted by YAP/TAZ modulation in governing the metabolic remodeling in physiological conditions or in somatic cell reprogramming. Nevertheless, on these bases, it is reasonable to also postulate a significant contribution of YAP/TAZ to the metabolic remodeling accompanying cellular reprogramming.

YAP/TAZ activation may regulate the switch from OXPHOS to glycolysis not only in tumor cells, but also in cell reprogramming, promoting glucose uptake and utilization by regulating the transcriptional rate of glycolytic enzymes, and glucose transporters [30,120]. This would be essential to sustain the increase in the proliferation rate of somatic cells undergoing reprogramming. Along with aerobic glycolysis, another hallmark of metabolic reprogramming is enhanced glutaminolysis, which serves to directly fuel the TCA cycle and produce key metabolites for cell growth, as well as ATP [126]. Many glutamine-metabolizing enzymes are transcriptionally governed by YAP [139,140,141] and upon myofibroblastic transdifferentiation, hepatic stellate cells undergo metabolic reprogramming activating aerobic glycolysis and increasing glutaminolysis in a YAP-mediated mechanism [71]. YAP/TAZ regulation of metabolic reprogramming also occurs through the interplay with mTOR [126].

Not only many metabolic-related genes, but also main transcription factors involved in the acquisition of the transient hyper-energetic state, like c-MYC, HIF-1α, NRF2 and OCT4 are directly or indirectly regulated by YAP [13].

Furthermore, YAP and TAZ regulate mitochondrial biogenesis and size. In both humans and *Drosophila*, YAP or Yorkie activation induces accelerated fusion, with the consequence of larger mitochondria [146]. On the contrary, forced mitochondrial fission is associated with inhibition of YAP and TAZ [147]. Thus, they might be relevant in mitochondrial remodeling upon somatic reprogramming.

Very recently, a novel role of YAP/TAZ in modulation of cell plasticity was discovered. Totaro et al. found that YAP/TAZ transcriptionally control autophagy by regulating autophagosomal degradation into autolysosomes, and linked this process to the YAP/TAZ-mediated dedifferentiation and acquisition of self-renewing properties of normal cells. The authors proposed that YAP/TAZ-driven autophagy represents a form of “cytoplasmic” reprogramming, a checkpoint ensuring that YAP/TAZ transcriptional rewiring in the nucleus goes hand in hand with the need for cytoplasmic and whole-cell renovation and restructuring [172].

Finally, YAP/TAZ can potentially exert profound effects on cell plasticity and metabolic reprogramming by affecting other processes like epigenetic modifications to chromatin structure [78,173] and post-transcriptional microRNA (miRNA) processing [174]. It is noteworthy that YAP/TAZ may influence the accessibility and activity of various target genes via chromatin structure alterations in association with chromatin-remodeling complex proteins such as nucleosome remodeling and deacetylase (NuRD), Switch/sucrose non-fermentable (SWI/SNF), nuclear receptor coactivator 6 (Ncoa6), Mediator, and GAGA factor [173]. MiRNAs have emerged not only as key regulators of metabolic homeostasis, but they are also important in maintaining the balance between differentiation and proliferation [175]. Post-transcriptional processing of miRNA is also regulated by YAP/TAZ via their modulation of Microprocessor activity [176], and Dicer activity [174].

## 6. Conclusions

YAP and TAZ have attracted considerable attention in the past decade for their multifaced functions, such as mechanotransduction, stemness, differentiation, proliferation, and tumorigenesis. Recently, the interplay between YAP/TAZ and metabolism has emerged. The findings paved the way for the elaboration of strategies in which modulation of YAP and TAZ may be used to reprogram differentiated cells into plastic progenitor cells or iPSCs able to functionally restore injured organs. As YAP and TAZ are often hyperactivated in human cancers, where they contribute to tumor development, their tissue activation for a long time may trigger detrimental effects. Moreover, hyperactivation of YAP/TAZ may not universally stimulate regeneration, as it causes different phenotypes in different organs. Thus, a better understanding of the role of YAP/TAZ in cell lineage fate determination would be advantageous for therapeutic applications in tissue engineering and regenerative medicine.
